# Study of angiotensin-converting enzyme insertion/deletion polymorphism, enzyme activity and oxidized low density lipoprotein in Western Iranians with atherosclerosis: a case-control study

**DOI:** 10.1186/s12872-019-1158-4

**Published:** 2019-08-01

**Authors:** Negar Nouryazdan, Glavizh Adibhesami, Mehdi Birjandi, Rouhollah Heydari, Banafsheh Yalameha, Gholamreza Shahsavari

**Affiliations:** 1 Department of Clinical Biochemistry, Faculty of Medicine, Lorestan University of Medical Sciences, Khorramabad, Iran; 2 Department of Biochemistry and Genetics, School of Medicine, Lorestan University of Medical Sciences, Khorramabad, Iran; 3 Nutritional Health Research Center, Lorestan University of Medical Sciences, Khorramabad, Iran; 4 Razi Herbal Medicines Research Center, Lorestan University of Medical Sciences, Khorramabad, Iran

**Keywords:** Coronary artery disease (CAD), Atherosclerosis, Renin-angiotensin system, Polymorphism

## Abstract

**Background:**

It has been indicated that Angiotensin-Converting Enzyme Insertion/Deletion (ACE I/D) polymorphism (rs4646994) could be regarded as a genetic factor that raises the risk of CAD through its impact on the activity of Angiotensin-Converting Enzyme (ACE) and angiotensin II level. The present study seeks to examine the relationship between ACE I/D polymorphism with the risk of atherosclerosis. Moreover, its potential effects on ACE activity and oxLDL level are investigated.

**Methods:**

In this study, 145 healthy individuals and 154 patients (143 males and 156 females) were selected among the subjects referred to Shahid Madani Hospital. Atherosclerosis was determined in all subjects with gold standard angiography. Blood samples were collected, used to isolate white blood cells (WBC) and serum separation. The DNA was extracted and the polymorphism was determined by polymerase chain reaction (PCR). The enzyme activity was measured using high-performance liquid chromatography (HPLC).

**Results:**

This study indicated that patients with atherosclerosis had higher levels of oxidized Low-Density Lipoprotein (oxLDL) and ACE activity (*P* < 0.05) as compared to controls. Although we found a significant association between ACE I/D polymorphism genotype and the allele with atherosclerosis in the male group, there were no association when the entire patient group was compared to the entire control group.

**Conclusion:**

Our study revealed the ACE I/D polymorphism of the ACE gene may not be an independent risk factor in the development of atherosclerosis and evaluation of ACE activity level is more important in evaluating the risk of disease. The researchers found no relation between ACE I/D polymorphism and atherosclerosis and also between types of genotype, ACE activity, and OxLDL level.

## Background

Even though therapeutic strategies concerning the deterrence of CAD have advanced, the rate of death induced by CAD is still rising. It is estimated by 2030 deaths due to CAD will be 23.4 million people. The most significant reason for CAD has been reported to be atherosclerotic coronary arteries [1, 2].

Atherosclerosis, as a fibroproliferative and inflammatory process, results from endothelial dysfunction induced chronic vascular damage [3]. A key step in initiating atherosclerosis is the LDL accumulation in the intima layer of the coronary artery [4]. LDL particles are converted to oxidized Low Density Lipoprotein (oxLDL) by reactive oxygen species (ROS) [5, 6]. Different risk factors have been identified in the occurrence of atherosclerosis including age, gender, high blood pressure, diabetes mellitus, hyperlipidemia, smoking, and obesity [7, 8].

Various published studies have emphasized that genetic factors could be involved in the initiation and progression of CAD [9]. For the first time, Rigat et al. discovered the ACE I/D polymorphism in their attempt to investigate the role of ACE gene in genetic control of plasma ACE level. They used this polymorphism as a genetic marker to examine the relationship between the type of polymorphism and the ACE concentration. Cambien et al. were the first to report the model for the genetic control of plasma ACE levels, based on the results of a family study. They showed 47% of the serum ACE variance was due to the allele effect of ACE I/D polymorphism. They also were the first to report the relationship of ACE I/D polymorphism with CAD.

Hence, we could consider insertion/ deletion (I/D) polymorphism of angiotensin-converting enzyme (ACE) gene as an effective genetic risk factors for CAD [10]. Many studies were conducted in different populations to investigate this association. We also decided to this for the first time in the Western Iran population. Maybe just say “The literature, however, is conflicting with some studies showing an association and others not.”

ACE is one of the components of the RAS and zinc-dependent metalloproteinase found widely in endothelial and epithelial cells. Moreover, the enzyme has been isolated from several sources including serum, lungs, seminal fluid, and plasma. ACE, as a key component in RAS, converts angiotensin I to angiotensin II Angiotensin II, increase the production of adhesion molecules and chemokines, stimulates LDL oxidation and foam cell formation in macrophages. Increased level of the ACE and subsequent ACE activity by raising the production of angiotensin II can lead to atherosclerosis [11–13].

A variety of investigations have reported the impact of ACE I/D polymorphism in several cardiovascular diseases including endothelial dysfunction, atherosclerosis, and heart failure. It has been displayed that there is a relationship between ACE I/D polymorphism and the alteration of ACE activity [14, 15]. Various studies have revealed that the ACE level is higher in patients with DD than subjects with II genotype, and those with ID genotype have a medium level [16]. The literature, however, is conflicting with some studies showing an association and others not [17, 18]. We have summarized some previous ACE I/D polymorphism studies in a table below. (Table 1).

**Table 1:** Summary of some previous ACE I/D polymorphism studies

Study	Region	Sample size		Genotype frequency in cases			Genotype frequency in controls			*P*-value
		Cases	controls	II	ID	DD	II	ID	DD	
1	Japan [19]	100	178	44	60	74	41	33	26	*P* < 0.01
2	North Iran [20]	369	141	21	69	51	95	180	94	*P* = 0.009
3	America [21]	169	132	83	69	17	72	51	9	*P* < 0.001
4	Asian populations [22]	378	313	133	174	71	114	129	70	*p* > 0.05
5	Italy [23]	236	158	20	118	98	20	71	67	*p* < 0.27
6	European population [24]	405	357	64	196	145	70	151	136	NS
7	Turkey [25]	218	107	26	64	128	18	47	42	*P* = 0.05
8	Southern Turkey [26]	176	131	6	81	89	14	66	51	*P* = 0.01
9	Caucasian [27]	540	349	120	262	158	75	173	101	*p* > 0.05
10	China [28]	114	157	50	46	18	62	63	32	*p* = 0.42
11	India [29]	203	212	61	88	54	59	80	73	*p* = 0.21

The purpose of the present study is the investigation of ACE I/D polymorphism distribution, measurement of ACE activity and oxLDL level and a number of biochemical factors**.** Furthermore, we examined the potential effect of ACE I/D polymorphism on ACE activity and the level of oxLDL in western Iran.

## Methods

### Study design and population

In this study, a total of 299 subjects (154 patients with atherosclerosis and 145 controls) who referred to Cardiology and Angiography Department of Shahid Madani Hospital, Khorramabad, Iran, between 2016 December and 2017 May were selected. Whether the participants had atherosclerosis or not was confirmed by the standard diagnostic angiography. In the case of plaque discernment inside arteries, participants divided into the patient’s group and, if the report showed normal coronary angiography, divided into the control group. The medical history of all subjects including age, sex, weight, the age of diagnosis, smoking, family history, hypertension, diabetes mellitus, drug abuse, and alcohol consumption were recorded. Patients with congenital heart disease, malignancy, chronic kidney disease, pulmonary obstruction, and steroid hormones consumption were excluded. Written informed consent was obtained from all participants. The study was approved by the Research Committee of Shahid Madani Hospital and the Ethics Committee of Lorestan University of Medical Sciences. Written informed consent was obtained from all subjects who participated in the study (code: LUMS.REC.1395.123). This study was administered in accordance with the Declaration of Helsinki and its following revisions.

### Biochemical measurements

The blood samples were collected from all subjects following overnight fasting into tubes without anticoagulant. The lipid profile was evaluated applying an auto-analyzer (BT-1000, USA).

Small dense Low Density Lipoprotein (SdLDL) was evaluated using the method by Hirano et al. as follows: 1. Precipitating serum lipoproteins with 1.044 g/ml density using heparin sodium salt and MnCl_2_, 2. incubating for 10 min at 37 °C, 3. placing in the ice bath for 15 min. 4, Collecting the supernatant by centrifugation at 15,000 rpm for 15 min at 4 °C and 5. Measuring LDL-cholesterol in the supernatant by LDL assay kit (Pishtazteb, Iran) [30].

**Fig. 1:**
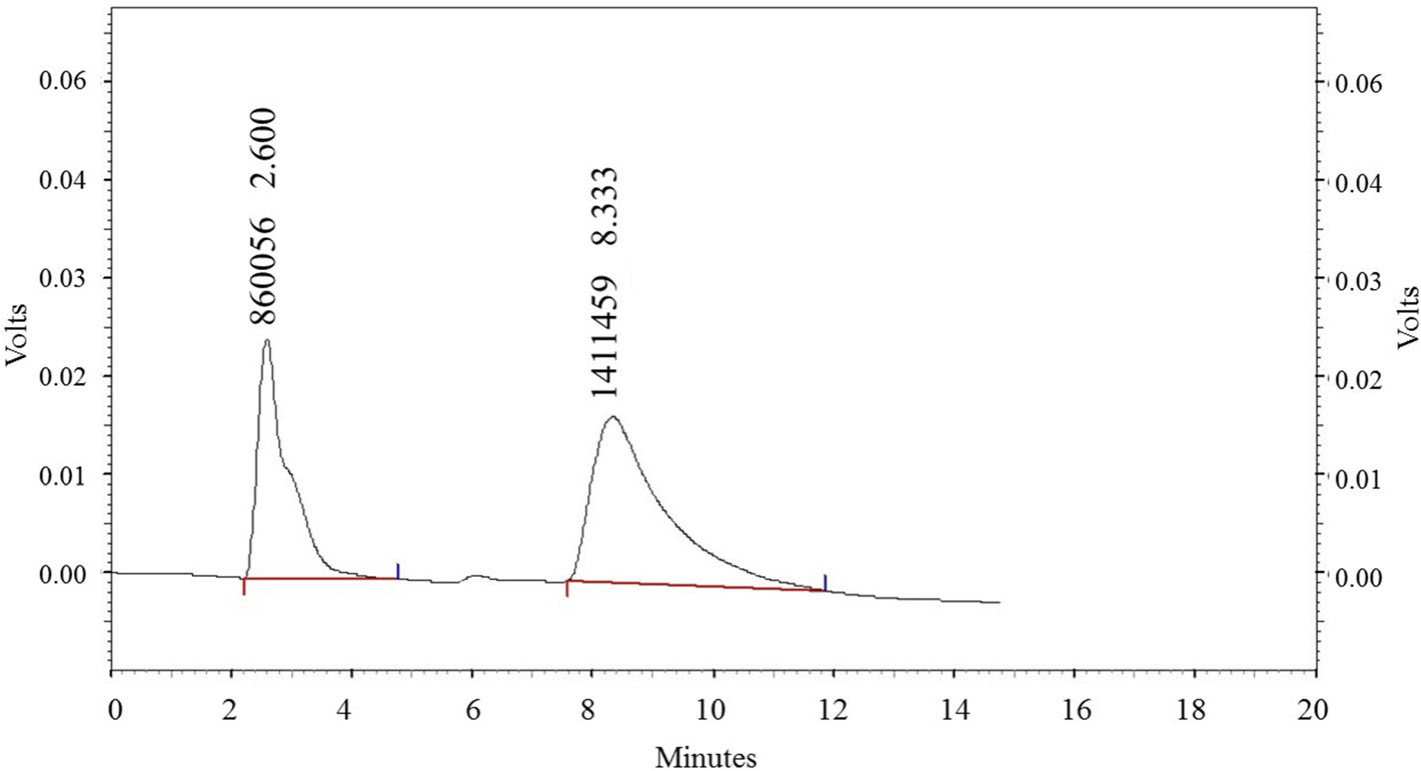
Standard sample of Hippuric Acid in HPLC

The measurement of oxLDL was performed using ELISA method according to the manufacturer protocol (Mercodia, Sweden). ACE activity was measured using the method by Horiuchi et al. In this measurement, 40 μl borate buffer containing 5.3 mM Hippuryl-L-Histidyl-Leucine (HHL) as a substrate, was added to 10 μl of serum, and then incubated for 30 min. The reaction was stopped by adding metaphosphoric acid, and was centrifuged for 5 min at 4000 rpm. Subsequently, 20 μl of supernatant was injected into the HPLC column. The amount of released hippuric acid was analyzed using HPLC (Shimadzu, Japan). The unit of enzyme activity is the amount of enzyme that can produce 1 μmol of hippuric acid at 37 °C for 1 min (Fig. 1) [31].

### Genotyping of the ACE polymorphism

**Fig. 2:**
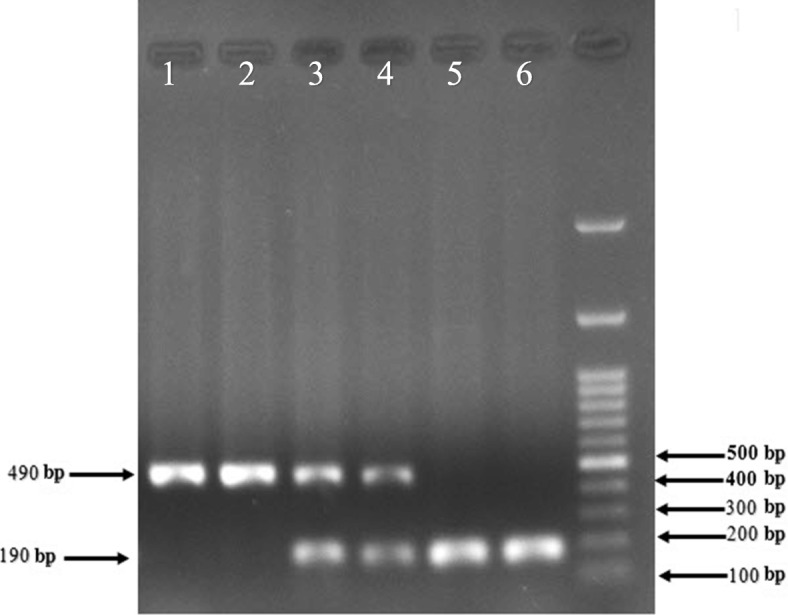
Gel electrophoresis of PCR amplified product of ACE gene I/D polymorphism

Genomic DNA was extracted from white blood cells with the DNA extraction kit (Yekta Tajhiz, Iran) based on the instruction of the manufacturer’s protocol. Genotyping for the ACE gene I/D polymorphism was performed using polymerase chain reaction (PCR) method and using two oligonucleotide primers, sense: 5′-CTGGAGACCACTCCCATCCTTTCT-3′ and antisense: 5′-GATGTGGCCATCACATTCGTCAGAT-3′ based on the flanking sequence of the insertion/deletion region on the ACE gene. The amplification was performed in a volume of 25 μl containing 50 ng template DNA, 10 μM of each primer, 2.5 μl 10X PCR buffer (Fermentas, Lithuania), 3 mM MgCl2, 200 μM each dNTP, and 1.5 units of Taq DNA polymerase (Fermentas, Lithuania). The PCR cycling conditions were as follows: initial denaturation at 94 °C for 5 min followed by 30 cycles of denaturation at 94 °C for 60 s, annealing at 59 °C for 60 s, extension at 72 °C for 2 min, and a final extension at 72 °C for 5 min. Amplification products were separated and sized by electrophoresis on a 2% agarose gel. The length of the D and I fragment alleles were 190 bp and 490 bp respectively (Fig. 2) [15].

To avoid DD mistyping, Second PCR was performed with a specific pair of primers that included sense: 5′-TGG GAC CAC AGC GCC CGC CAC TAC-3′ and antisense: 5′-TCG CCA GCC CTC CCA TGC CCA TAA-3′, in 25 μl reaction mixture volume on DD genotype samples. PCR conditions were identical except for the annealing temperature of 57 °C. The presence of I allele resulted in a 335 bp PCR product. All samples with DD genotype were re-genotyped, which I allele was detected in 8 samples.

### Statistical analysis

The normality of data was tested by the use of the Kolmogorov-Smirnov test. The data of the numerical variables are presented as mean ± SD. Accordingly, t-test was used to compare continuous data, and Chi-square test was used to test the qualitative variables. Frequencies of genotype and allele was tested using Chi-square test and association with disease was tested using logistic regression. We used ANOVA and tukey’s test (for post-hoc analysis) for intra-group comparison of ACE activity and oxLDL level. Multivariate logistic regression analysis was used for testing the independent association of various variables. Data were analyzed with SPSS software version 16 (SPSS Inc., Chicago, IL, USA). *P*-values less than 0.05 were regarded to be statistically significant.

## Results

### Baseline characteristics of the study population

The baseline characteristics of the 299 participants have been presented in Table 2. The results of the present study revealed a significant difference between age (*P* < 0.001), weight (*P* = 0.045) and diagnosis age (Age of diagnosis the heart disease) (*P* < 0.001) of the patient and control groups. Remarkably, there was not any significant difference between height (*P* = 0.112), BMI (*P* = 0.390), systolic blood pressure (*P* = 0.140) or diastolic blood pressure (*P* = 0.147) in the two groups.

**Table 2:** Baseline characteristics of the study population, analysed by t-test

Characteristics	Control (*N* = 145) (mean ± SD)	Patient (*N* = 154) (mean ± SD)	*P*-Value
Age (years)	55.57 ± 9.98	63.39 ± 10.65	< 0.001*
Weigh (kg)	73.02 ± 17.18	86.15 ± 78.54	0.045*
Height (cm)	163.61 ± 11.97	165.57 ± 9.08	0.112
BMI (kg/m2)	28.81 ± 24.72	31.25 ± 24.37	0.390
Age of diagnosis (years)	54.26 ± 90.53	61.58 ± 10.84	< 0.001*
Systolic BP (mmHg)	130.86 ± 13.59	135.01 ± 31.84	0.140
Diastolic BP (mmHg)	80.39 ± 10.15	82.09 ± 10.11	0.147

*BP* Blood Pressure, *BMI* Body Mass Index

*Statistically significant (*p* < 0.05)

The biochemical characteristics of the study subjects have been indicated in Table 3. A significant difference was found between TC (*P* < 0.001), TG (*P* = 0.006), HDL (*P* = 0.035), LDL (*P* < 0.001), Sd-LDL (*P* < 0.001), Ox-LDL (*P* < 0.001), serum glucose (*P* < 0.001) and ACE activity (*P* = 0.026) between the patient and control groups. Furthermore, we found a significant difference between the family history of CAD, diabetes mellitus, cigarette smoking and hypertension between the two groups.

**Table 3:** Biochemical characteristics of study population, analysed by t-test and Chi-square

Variable	Control (*N* = 145) (mean ± SD)	Patient(*N* = 154) (mean ± SD)	*P*-Value
FBS (mg/dl)	94.89 ± 25.93	120.76 ± 52.63	* < 0.001
TC (mg/dl)	146.69 ± 37.71	177.38 ± 58.91	* < 0.001
TG (mg/dl)	128.43 ± 58.79	151.15 ± 80.49	0/006*
HDL (mg/dl)	40.32 ± 14.83	37.3 ± 19.13	0/035*
LDL (mg/dl)	77.67 ± 27.40	94.03 ± 34.32	* < 0.001
Sd-LDL (mg/dl)	15.58 ± 6.14	32.25 ± 11.54	* < 0.001
Ox-LDL (U/L)	35.41 ± 8.91	40.71 ± 11.34	* < 0.001
ACE activity (μmol/min.l)	39.68 ± 37.08	49.69 ± 40.33	0/026*
Family history of CAD n (%)^a^	53(36.6%)	102(66.2%)	* < 0.001
Hypertension n (%)^b^	61(42.1%)	87(56.5%)	0.009
Cigarette smoking n (%)^c^	18(12.4%)	59(38.3%)	* < 0.001
Diabetes mellitus n (%)^d^	15(10.3%)	39(25.3%)	* < 0.001

*TC* Total Cholesterol, *TG* Triglycerides, *HDL* High Density Lipoprotein, *LDL* Low Density Lipoprotein, *Sd-LDL* Small Dense LDL, *Ox-LDL* Oxidized Low-Density Lipoprotein, *ACE* Angiotensin-Converting Enzyme

*Statistically significant (*p* < 0.05)

^a^People who have had a history of CAD in their family

^b^People with a history of hypertension

^c^People who have had a history of smoking in their lives, Previously smokers and current smoker

^d^People with diabetes mellitus

### Genotype distribution and genotype frequencies

Genotype distribution did not deviate from Hardy-Weinberg equilibrium (patients (*P* = 0.2), controls (*P* = 0.08)). The results of the genotyping of the ACE polymorphism (Fig. 2) and its relationship with atherosclerosis have been presented in Table 4. In the present study, we did not find any significant difference between genotype and allele frequency in the patient and control groups. All the study groups and subgroups were separated by sex, and were subsequently analysed. We found a significant relation between ACE I/D polymorphism genotypes and the alleles with atherosclerosis in the male (81 patients, 62 control) group but there was no significant association in the female group (*P*-value = 0.02 for the male group, *P* value = 0.879 for the female group) (Table 5).

**Table 4:** Genotype and allele type of ACE I/D polymorphism in the patients and control groups, analysed by Logistic regression

Genotype	Controls (*N* = 145), n (%) (62 males,83 females)	Patients (*N* = 154), n (%) (81males,73 females)	Total, n (%)	OR (95% CI)	*P*-Value
II	29(20%)	20(13%)	49 (16.4%)	Ref.	
ID	70(48.3%)	81(52.6%)	151(50.5%)	1.67 (0.3–873.225)	0.121
DD	46(31.7%)	53(34.4%)	99(33.1%)	1.67 (0.3–835.341)	0.147
Allele					
I	128(44.1%)	121(39.3%)	249(41.6%)	Ref.	
D	162(55.9%)	187(60.7%)	349(58.4%)	1.221 (0.882–1.691)	0.229

*ACE* Angiotensin-Converting Enzyme, *CI* Confidence Interval, *I/D* Insertion/Deletion, *OR* Odds Ratio

**Table 5:** Genotype and allele type of ACE I/D polymorphism in the patients and control groups by gender, analysed by Chi-square

Genotype	Controls (*N* = 145) n (%)	Patients (*N* = 154) n (%)	Total, n (%)	*P*-Value
Female				
II	13(15.7%)	13(17.8%)	26(16.7%)	0.879
ID	41(49.4%)	37(50.7%)	78(50%)	
DD	29(34.9%)	23(31.5%)	52(33.3%)	
Allele				
I	67(40.4%)	63(43.2%)	130(41.6%)	0.65
D	99(59.6%)	83(56.8%)	182(58.4%)	
Male				
II	16(25.8%)	7(8.6%)	23(16.1%)	0.02
ID	29(46.8%)	44(54.3%)	73(51%)	
DD	17(27.4%)	30(37%)	47(32.9%)	
Allele				
I	61(49.2%)	58(35.8%)	119(41.6%)	0.03
D	63(50.8%)	104(64.2%)	167(58.4%)	

Patients with II and ID genotypes showed higher ACE activity as compared to controls with the same alleles (*P* < 0.05), but there was no significant association between ACE activity and DD genotype (*P* = 0.646) in the two groups. In contrast with the control group, the difference between the levels of ACE activity in different genotypes of the patient group was not statistically significant. The results indicated that in the control group, the level of enzyme activity of the DD genotype was significant (*p* < 0.05) compared to ID and II genotypes. No significant association was found between the other genotypes. The results also indicated that the relationship between genotype interaction and enzyme activity of ACE in increasing the chances of developing atherosclerosis was not significant.

Patients with DD and ID genotype showed higher oxLDL level as compared to controls with DD and ID genotype (*P* < 0.05), but there was no important distinction between oxLDL level and II genotypes (*P* = 0.156) in the two groups.

No specific pattern was found for the relationship between type of genotype and oxLDL level (Table 6). The present study revealed that the interaction effect of ID (*P* = 0.713), DD (*P* = 0.142) and II (*P* = 0.125) genotypes and ACE activity on oxLDL levels were not statistically significant.

**Table 6:** Comparison of ACE activity and oxLDL level between different ACE I/D polymorphism genotype and groups, analysed by t-test and ANOVA

Genotype	Controls	Patients	*P*-Value
ACE activity (μmol/min.l)			
II	30.67 ± 26.82	56.15 ± 46.11	0.034
ID	36.31 ± 34.69	45.03 ± 38.77	0.002
DD	*50.50 ± 43.77	54.39 ± 40.29	0.646
*P*-value	0.037	0.181	
oxLDL level (U/L)			
II	37.34 ± 9.68	41.15 ± 8.12	0.156
ID	34.85 ± 8.5	40.19 ± 11.82	0.002
DD	35.04 ± 9.17	41.33 ± 11.76	0.004
*P*-value	0.449	0.683	

*Significant differences in the enzyme activity level of DD genotype compared to genotype II and ID at 0.05

Using the multivariable logistic regression, the effects of ACE I/D polymorphism genotypes simultaneously with regard to age, sex, history of blood pressure, smoking and CAD, were simultaneously examined. The results indicated no significant relationship between genotype and the risk of developing atherosclerosis. In terms of age, the risk of developing a disease is increased by 0.8% per 1-year-old increase, which is statistically significant (*P* < 0.001) (CI = 1.05–1.11). As expected the risk of atherosclerosis in patients with a history of cardiovascular disease is 3.56 times more than those without a history of the same disease. (*P* < 0.001) (CI = 2–6.33) (Table 7).

**Table 7:** Effect of ACE I/D, genotypes, age, gender, history of hypertension, smoking, and CAD on atherosclerosis using multivariate logistic regression

Genotypes	Group	OR(95% CI)	*P*-Value^a^
ACE I/D	II	Ref	
	ID	1.95(0.88–4.31)	0.99
	DD	1.67(0.73–3.83)	0.222
Gender	Women	Ref.	
	Men	1.7(0.91–3.16)	0.092
Hypertension	No	Ref.	
	Yes	1.23(0.68–2.32)	0.485
Cigarette smoking	No	Ref.	
	Yes	4.13(2.05–9.05)	< 0.001
Family history of CAD	No	Ref.	
	Yes	3.56(2–6.33)	< 0.001
Age (years)		1.08(1.05–1.11)	< 0.001

^a^Adjusted for age, Gender, Hypertension, Cigarette smoking and Family history of CAD

## Discussion

After reorganization of ACE I/D polymorphism as a genetic marker for cardiovascular disease, many investigations were done to find such a genetic risk relationship. This study was planned to investigate the association between ACE I/D polymorphism and the risk of atherosclerosis, and the effect of genotypes on ACE activity. We also interested in to study the difference of ACE activity, oxLDL level, and a number of biochemical characteristics between two groups. Distributions of the genotypes frequency of ACE I/D polymorphism in our study were found to be I/I (20%), I/D (48.3%), and DD (31.7%) in the control group and I/I (13%), I/D (50.5%), and DD (33.1%) in the patient group. In the present study, neither genotype nor allele frequency was remarkably different between the control and patient groups. This finding confirms the results of certain studies previously conducted that will be discussed below.

Many studies showed that genetic factors may play a role in the progression of cardiovascular diseases. Among these genetic factors, the ACE I/D gene polymorphism has been a recurrent subject in a variety of studies [32]. The ACE converts angiotensin into active octapeptide, called angiotensin II, which is the main active component in the RAS and has been known as an atherogenic factor [33]. Various studies have indicated that ACE I/D polymorphism could be as a risk factor for CAD, MI, and cardiomyopathy [34].

The relationship between DD genotype and CAD has been examined in several studies [35]. It has been found out that DD genotype is a risk factor for the development of atherosclerosis in carotid arteries in the Chinese, Australian, and Asian Indian populations [36, 37]. Moreover, the D allele has a role in the incidence of CAD by elevating the levels of ACE in several populations such as Turkish [38].

In accordance with the result obtained from our study, some studies previously carried out found no relationship between ACE I/D polymorphism and incidence of CAD. Studies carried out by Jeunemaitre et al. and Ferrieres et al. indicated that ACE I/D polymorphism could not play a role in the occurrence of CAD in the Caucasian and European populations [24, 39]. A meta-analysis study was conducted on 118 different studies, including 43.733 patients and 82.606 healthy individuals. Their results indicated that ACE I/D polymorphism is correlated with the increase in the risk of sever CAD [40].

The ACE enzyme plays an important role in the RAS by converting angiotensin I to angiotensin II. Angiotensin II has been shown to have atherogenic properties. Increased activity of the ACE enzyme can contribute to an increased risk of disease by raising angiotensin II production. Therefore, the level of ACE activity that may be affected by various factors, including genetic factors, can be considered as a risk for the disease [41]. Many studies have shown the relationship between ACE serum levels and ACE I/D polymorphism in different population [42–44]. In our study the level of ACE activity in patients with II + ID and DD genotypes was significantly higher than in control (II + ID and DD) that can consider as an important risk factor for disease in a study conducted by Sahin et al. (2015) on the population of Turkey, an increase was observed in ACE activity in the patient group compared to the control group. Furthermore, DD genotype was more prevalent among patients in their research [41]. It seems that evaluation of ACE activity level in comparison with ACEI/D polymorphism genotypes is more important in evaluating the risk of disease.

Various pieces of evidence indicated that LDL peroxidation is one of the most important risk factors for atherosclerosis. In fact, changes in LDL oxidation seem to be the most important trigger for the development of atherosclerosis. Researchers have indicated that angiotensin 2, a product of the ACE, could play a role in increasing the absorption and oxidation of LDL [45–47].

For the first time we investigated the relationship between ACE I/D polymorphism and oxLDL level. In the present study a significant difference was observed in the level of oxLDL in DD + ID and DD genotypes in the patient group in contrast with the control group. No significant correlation was found between the simultaneous effect of ACE activity and ACE I/D polymorphism on oxLDL levels. Investigations by Shimada et al. showed high level of oxLDL in patient with coronary artery disease than controls [48]. In addition, our biochemical parameter’s results such as sdLDL, lipid profiles, blood pressure, FBS, and patient records were consistent with many previous findings [49].

## Limitations

The limitations about this study include: first, in this study, we only collected samples from several western provinces in Iran, which, due to the genetic diversity and racial differences in different populations in Iran, this investigation could study a larger population in Iran, which may have different outcomes in different ethnic groups. Secondly, we did not find any relationship between different ACE I/D polymorphism genotypes and atherosclerosis in the control and patient groups, so other factors such as confirmation of genotyping results should be considered. Third, this study did not investigate the effects of oxidative stress, which, can increase the risk of heart disease. So, there are some other factors that can be very affected in this way such as environmental pollution factors.

## Conclusion

In this study, we observed a considerable association between ACE I/D polymorphism and atherosclerosis in the male group, but there was no relationship between different ACE I/D polymorphism genotypes and atherosclerosis in the control and patient groups. This fact that the level of ACE activity and oxLDL were significantly higher in the patient group than the control group indicates the role of these two factors in increasing the risk of atherosclerosis. However, we did not find any evidence that ACE I/D polymorphism could effect on the level of enzyme activity and oxLDL. Despite the small sample size, this investigation showed the study of ACE I/D polymorphism did not proper for the prediction of atherosclerosis.

## Data Availability

The datasets generated and/or analyzed during the current study are available from the corresponding author on reasonable request.

## Abbreviations

ACE I/D: Angiotensin-converting enzyme insertion/deletion
CAD: Coronary artery disease
HDL: High-density lipoprotein
LDL: Low-density lipoprotein
OxLDL: Oxidized LDL
RAS: Renin-angiotensin system
SdLDL: Small dense LDL
